# Establishing a Mentoring Scheme for Psychiatrists in the South West, UK

**DOI:** 10.1192/bjo.2025.10277

**Published:** 2025-06-20

**Authors:** Emily Thomas, Heena Hargovan, Claudia Calciu, Katie Ewart

**Affiliations:** 1 Herefordshire and Worcestershie Health and Care NHS Trust, Worcester, United Kingdom; 2 Gloucestershire Heath and Care NHS Foundation Trust, Gloucester, United Kingdom

## Abstract

**Aims:** To establish a formalised process for mentoring to be accessed by psychiatrists of all grades within a mental health trust in Gloucestershire, UK. Mentoring can contribute to career success, higher self-esteem and job satisfaction for mentees and improve performance and job satisfaction for mentors.^[1]^ This in turn fosters innovation and development as part of the culture within the host organisation.^[2]^ We anticipate the creation of a mentoring scheme for psychiatrists will promote these improvements for individuals in the short term and the organisation in the longer term.

^[1]^ The General Medical Council (GMC). The mentoring toolkit: Setting up a formal mentoring scheme. August 2024. ^[2]^ Coaching and mentoring. The NHS Leadership academy. https://www.leadershipacademy.nhs.uk/programmes/coaching-and-mentoring/. Accessed November 13, 2024.

**Methods:** The European Mentoring and Coaching Centre defines mentoring as ‘a learning relationship, involving the sharing of skills, knowledge, and expertise between a mentor and mentee through developmental conversations, experience sharing, and role modelling’.

Mentoring is promoted as part of *Good medical practice* by the GMC. The Royal College of Psychiatrists highlights its importance for all doctors, especially around periods of transition and to aid in retention and resilience of the workforce.

Gloucestershire Health and Care NHS Foundation trust appointed an experienced psychiatrist as a mentoring lead in 2022 with ambitions to develop a scheme in the trust. A steering group was formed to develop this idea and oversee its inception.

An electronic survey to medical colleagues in psychiatry identified significant interest for a formal mentoring scheme.

Exploration of best practice in adjacent trusts and a literature search developed the organisational process and allocation procedure of pairings. Centralising requests and the use of mentor biographies allowed for the mentor-mentee matching, and a database created.

The scheme is modelled on traditional mentorship, with anticipation of senior clinicians acting as mentors to trainees or more junior colleagues in all cases, unless peer to peer mentoring is requested.

**Results:** Initially, 8 mentor/mentee matches were made, with a further 7 pairings captured with steering group oversight. Feedback was unanimously positive with praise for the ease of the process and benefits gained from the relationship exchange from both mentors and mentees.

**Conclusion:** The scheme has an email address for centralising enquiries and is signposted via a flyer across trust working areas and as part of induction for all new doctors in Psychiatry. 4 CPD talks have been delivered to the medical academic programme and next steps include an annual newsletter, development of bitesize training for mentors and a video to further raise the profile of the scheme. Limitations of the scheme are acknowledged given moderate size of the trust and small pool of available mentors.

